# Emerging nano-scale delivery systems for the treatment of osteoporosis

**DOI:** 10.1186/s40824-023-00413-7

**Published:** 2023-07-13

**Authors:** Anoop Puthiyoth Dayanandan, Woong Jin Cho, Hyemin Kang, Alvin Bacero Bello, Byoung Ju Kim, Yoshie Arai, Soo-Hong Lee

**Affiliations:** 1 Department of Biomedical Engineering, Dongguk University, Seoul, 04620 Republic of Korea; 2 ATEMs, Seoul, 05836 Republic of Korea

**Keywords:** Osteoporosis, Nano-based materials, Nano-based delivery system

## Abstract

Osteoporosis is a pathological condition characterized by an accelerated bone resorption rate, resulting in decreased bone density and increased susceptibility to fractures, particularly among the elderly population. While conventional treatments for osteoporosis have shown efficacy, they are associated with certain limitations, including limited drug bioavailability, non-specific administration, and the occurrence of adverse effects. In recent years, nanoparticle-based drug delivery systems have emerged as a promising approach for managing osteoporosis. Nanoparticles possess unique physicochemical properties, such as a small size, large surface area-to-volume ratio, and tunable surface characteristics, which enable them to overcome the limitations of conventional therapies. These nanoparticles offer several advantages, including enhanced drug stability, controlled release kinetics, targeted bone tissue delivery, and improved drug bioavailability. This comprehensive review aims to provide insights into the recent advancements in nanoparticle-based therapy for osteoporosis. It elucidates the various types of nanoparticles employed in this context, including silica, polymeric, solid lipid, and metallic nanoparticles, along with their specific processing techniques and inherent properties that render them suitable as potential drug carriers for osteoporosis treatment. Furthermore, this review discusses the challenges and future suggestions associated with the development and translation of nanoparticle drug delivery systems for clinical use. These challenges encompass issues such as scalability, safety assessment, and regulatory considerations. However, despite these challenges, the utilization of nanoparticle-based drug delivery systems holds immense promise in revolutionizing the field of osteoporosis management by enabling more effective and targeted therapies, ultimately leading to improved patient outcomes.

**Supplementary Information:**

The online version contains supplementary material available at 10.1186/s40824-023-00413-7.

## Introduction

Bone, being a metabolically dynamic tissue, serves as the foundational framework of the human skeletal system. It encompasses three fundamental functions of paramount significance. Firstly, bone acts as a reservoir for diverse elements, including magnesium, phosphate, and bicarbonate, thus playing a pivotal role in the maintenance of calcium homeostasis [1]. Secondly, it assumes a critical role in providing protection against internal injuries. Lastly, bone is essential for adult hematopoiesis, the process of blood cell formation [2]. To accomplish these functions, bone tissue must uphold a delicate equilibrium between its resorption and formation, known as bone remodeling [3].

Disruption of the intricate bone remodeling processes can give rise to a spectrum of disorders, leading to detrimental effects on the skeletal system and resulting in compromised mobility and potentially life-threatening consequences. Notable skeletal disorders include osteosarcoma, non-union bone defects, osteoarthritis, osteoporosis (OP), and bone metastatic cancers [4, 5]. Among these, OP is the most prevalent metabolic bone disease that primarily affects the elderly and postmenopausal women [6, 7]. OP is described by the World Health Organization as a “progressive systemic skeletal disease characterized by low bone mass and microarchitectural deterioration of the bone tissue, resulting in increased bone fragility and susceptibility to fracture” [8]. OP predominantly affects vulnerable regions of the skeleton, leading to decreased bone density [9, 10]. Moreover, OP induces structural weakening of the vertebrae, hip joint, and carpal bones due to aberrant remodeling of trabecular and cortical bones [6, 11, 12].

Multiple research endeavors have been dedicated to developing treatment options for OP. However, striking a delicate balance between drug efficacies and minimizing adverse effects remains a formidable challenge. There exists an imperative to explore innovative approaches that enable the safe and efficient delivery of drugs to the bone tissue, thereby enhancing drug efficacy while mitigating side effects [13, 14]. To address the immediate needs of OP patients, an array of interventions, including amputation surgery, chemotherapy, radiation therapy, and drug injection, have been employed [13]. While effective in managing OP, these methods are not suitable for long-term and localized therapies, and they are associated with a range of side effects. These include broad tissue distribution [15], suboptimal targeting efficiency leading to off-target effects [16], limited drug half-life, inadequate bioavailability [14], and inadequate availability of bone graft sources [17]. Furthermore, these approaches pose risks of infection and uncontrolled drug release [17, 18]. To mitigate these side effects, drug delivery systems (DDSs) based on biocompatible materials have been developed. Table 1 provides an overview of materials employed in DDSs for OP treatment. Although these systems have demonstrated enhanced therapeutic efficacy in addressing OP, further research is required to overcome the challenges elucidated in Table 1.

**Table 1:** **Pros and cons of various drugs used in the treatment of osteoporosis**. Conventional drug delivery systems for osteoporosis have limitations including low selectivity, poor bioavailability, short half-life, potential side effects, invasive administration routes, lack of targeted delivery, and limited control over drug release. Addressing these limitations is a current area of research for improved therapeutic approaches

Ref. No.	Material	Character	Pros	Cons
[44]	Statin	Inhibitors of hydroxymethylglutaryl-CoA reductase (cholesterol-lowering)	Stimulates bone formation by upregulating bone morphogenic protein-2 (BMP-2) in bone cells	High dosage can increase OP risk
[45, 46]	Strontium-related compound	Alkaline earth metal (highly reactive)	Decreases fracture risk by increasing bone stiffness, stimulates osteoblastic activity, and inhibits bone absorption by osteoclasts (OC)	High dosage can cause several side effects
[47]	Ceria	An oxide of the rare-earth metal cerium	It has oxidative activity in the low-pH microenvironment generated by OCs and upregulates reactive oxygen species, which can decrease the viability of OCs.	Long-term use can induce cytotoxicity and genetic modification
[48]	W9 peptide	An antagonist of receptor activator for nuclear factor-κappa beta ligand (RANKL) and tumor necrosis factor-α (TNF-α)	Inhibits osteoclastogenesis and OC activity by suppressing autophagy and promoting apoptosis	The peptide can self-aggregate and is easily degraded in the body
[49]	Calcitonin -related peptide	A member of the calcitonin family of peptides that is found in the peripheral and central nervous systems	Activates the proliferation of BMSCs and promotes their osteogenic differentiation in physiological conditions	The peptide can self-aggregate and is easily degraded in the body
[50]	Raloxifene	A member of the synthetic compound for selective estrogen receptor modulators	Decreases accelerated bone turnover, increases bone mineral density, and recovers lost bone tissue in postmenopausal OP; decreases the risk of fractures in patients with previous vertebral fractures	Long-term use can cause side effects
[51]	Bisphosphonate	Inorganic pyrophosphate; have two phosphonate [PO(OH)_2_] groups	Can target bone tissue and reduce the activity of OCs.	High dosage can cause esophageal cancer and osteonecrosis
[52]	Salmon calcitonin	Synthetic calcitonin hormone	Inhibits bone resorption, prevents bone loss, and decreases fracture risk	High dosage can cause several side effects, and it is easily degraded in the body
[53]	Nucleic acid (RNA or DNA)	Used for gene therapy (gene editing and silencing)	Highly effective and can cure genetic diseases	Easily degraded by endogenous nucleases
[54]	Estrogen	Regulates the growth, differentiation, and functions of various tissues in women and maintains the female reproductive physiology	Direct effect on OCs and OBs to modulate trabecular versus cortical bone mass	Long-term use can cause side effects
[55]	Curcumin	Effective antioxidant	Stimulates the apoptosis of OCs, inhibits bone resorption, and suppresses osteoclastogenesis through the RANKL pathway	Low water solubility (lipid-soluble)
[56]	Icariin	Pharmacologically active flavonoid glycoside	Promotes bone formation by stimulating the osteogenic differentiation of BMSCs while inhibiting their osteoclastogenic differentiation	Low water solubility and low bioavailability after oral administration
[57]	Quercetin	Plant-derived flavonoid	Inhibits RANKL-mediated osteoclastogenesis, OB apoptosis, oxidative-stress generation, and inflammatory responses while promoting osteogenesis, angiogenesis, antioxidant expression, adipocyte apoptosis, and OC apoptosis	Low water solubility and low bioavailability after oral administration
[58]	Odanacatib	Inhibitor of cathepsin K (related to bone resorption)	Increases bone mineral density, inhibits bone resorption of OCs and their differentiation	Low water solubility and low bioavailability after oral administration

BMSC: Bone-marrow–derived mesenchymal stem cells; RANKL: Receptor activator of nuclear factor kappa beta

Nanomedicine, an expeditiously progressing discipline within the realm of material science, presents a plethora of advantages such as drug side effect reduction and biomimicking capabilities [19–21]. The exploration of nanomedicine has been underway since the late 1970s, and after the late 1980s, three pivotal strategies have emerged as primary drivers in the domain of nano-based drug delivery systems (DDSs): (1) PEGylation, (2) active targeting, and (3) the enhanced permeation and retention (EPR) effect [22]. PEGylation, a technique involving the conjugation of poly(ethylene glycol) to drugs, was initially investigated in the late 1960s to enhance drug stability and prolong circulation time within the organism [23]. Active targeting, which enables the attachment of targeting molecules such as ligands and antibodies to drugs, has been feasible since the late 1950s [24]. The discovery of the EPR effect in 1984 by Hiroshi Maeda of Kumamoto University facilitated the formation of polymer-conjugated DDSs through diverse biomaterial conjugations, thereby harnessing the phenomenon [25]. These three strategies have significantly propelled the advancement of research in nano-based DDSs, delivering substantial advantages in the field of drug delivery. However, further investigation is indispensable to effectively address the challenges associated with these systems.

Nanoparticles (NPs) are being used as an alternative method for bone-targeted treatment. Because of their small size and similarity to the components found in tissues [26], the nanoparticle materials can be delivered to specific tissues, organelles, or cells where the medicine will be released [27]. The advantages of NPs 1are that they provide a large capacity of the drug concerning size [28, 29], improve solubility [30], provide drug stability [31], reduce adverse effects [32], and improve transport for drug internalization in specific organelles [33]. Another advantage is that the NPs, which are composed of calcium phosphate, gold, and nanodiamonds, can help to activate functions in the cells, improve mineralization, and stimulate bone growth [32]. Thus, nanotechnology can overcome the limitations of conventional bone therapy, such as adverse effects and poor penetration to skeletal lesions.

The employment of nano-based DDSs offers the potential to enhance drug solubility and stability within the human body by conferring a biocompatible protective shield [34–37]. Moreover, the drug-loading capacity can be meticulously controlled through size adjustments of the DDSs [38, 39]. These DDSs possess inherent characteristics that facilitate the regulation of drug-release rates [40–43]. Recent investigations have explored diverse nanomaterials in the pursuit of advancing drug delivery, and in this review, we aim to present a concise overview of current research endeavors pertaining to nano-based DDSs.

## Nano-based approaches for the treatment of OP

Osteoporosis is a systemic skeletal disorder that exhibits diminished bone strength and an elevated susceptibility to fractures during routine activities. This condition is characterized by a reduction in bone mineral density (BMD) and deterioration of the bone microarchitecture, leading to compromised structural integrity. OP not only undermines the physical well-being of individuals but also significantly impacts their overall quality of life [59]. In a state of normal health, the dynamic balance between OCs and OBs ensures efficient bone remodeling, facilitating the repair of microdamage caused by routine activities. OCs are responsible for the breakdown of bone tissue, while OBs play a vital role in its formation. However, in the case of OP, there is an imbalance in this process, favoring OC activity over OB function. Consequently, the trabecular region, in particular, experiences a loss of bone mass. This reduction in trabecular connectivity renders the bone more susceptible to brittleness and significantly heightens the risk of fractures [60].

**Fig. 1:**
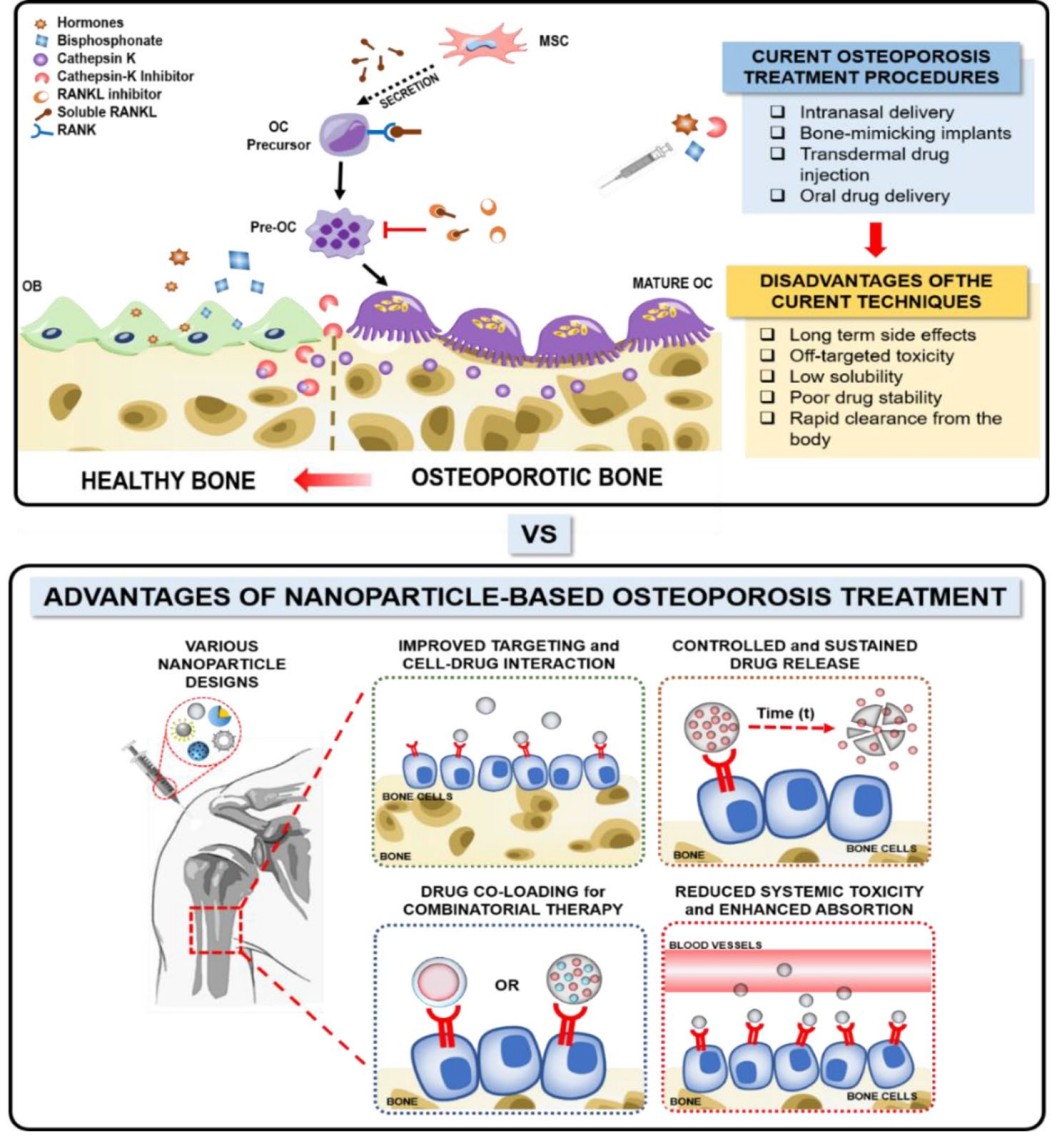
**A schematic illustration depicting the disadvantages and advantages between conventional treatment methods and nanoscale-based treatment in addressing OP**. Drugs like bisphosphates and hormones have been used to treat osteoporosis with treatment approaches like oral and intranasal delivery; however, these exhibit numerous drawbacks like long-term side effects, low drug solubility, and poor drug stability. To overcome these limitations, employing a nano-based DDS emerges as a potential solution. Nano-based DDSs provide various advantages like controlled and sustained drug release, improved targeting, and reduced systemic toxicity.MSC: Mesenchymal stem cells; OB: Osteoblast; OC: Osteoclast

Considering the complexities associated with OP, alternative strategies involving biomaterials, particularly nanomaterials, have been explored to address this condition and promote bone regeneration. In this context, nano-based DDSs hold promise as a potential solution. Therefore, to improve the limitations posed by the conventional DDSs, nano-based DDSs could offer a potential solution (Fig. 1).

Nanomaterials are characterized as materials with dimensions ranging from 1 to 100 nanometers. In this submicron scale, the properties of materials undergo significant changes attributed to quantum effects and the amplified surface-to-volume ratio [61]. These unique characteristics make nanomaterials particularly promising for applications in the field of OP treatment and bone tissue engineering. Some of the main approaches will be described next.

### Inorganic nanomaterials

Nanomaterials have garnered significant attention in the realm of bone repair owing to their distinctive osteoconductive properties. They exhibit potential as carriers for localized drug delivery, enabling the administration of diverse therapeutic agents. These nanomaterials possess customizable pore structures, adjustable pore sizes, and large surface areas, rendering them suitable for accommodating therapeutic entities ranging from proteins and genes to small molecular drugs. Moreover, these NPs can be further modified to regulate drug loading and influence the behavior of bone cells and immune cells [62–65].

#### Silica NPs

Silica, a biocompatible element, exhibits numerous biological advantages and has demonstrated the potential to enhance the osteoconductivity of hydroxyapatite (HA) bone scaffolds. Nevertheless, the underlying mechanism by which silica modulates skeletal development remains largely unexplored [66, 67]. To synthesize silica NPs, the sol-gel method was employed utilizing a tetraethoxysilane (TEOS) solution. The sol-gel process involved the hydrolysis of TEOS molecules, followed by condensation reactions facilitated by water and alcohol, which promoted nucleation and subsequent growth. As a result, the conversion of TEOS facilitated the formation of a silica network, ultimately yielding silica-based NPs [68].

Beck et al. investigated the in vitro effects of 50 nm fluorescent silica-based NPs on the differentiation of OBs and OCs. The study findings unveiled that these silica NPs exhibited the capacity to diminish the formation of multinucleated cells (RAW 264.7) in a concentration-dependent manner (25–100 µg/mL) without inducing apoptosis in OC precursors. Notably, the silica-based NPs also stimulated the differentiation of pre-OBs (MC3T3-E1) into mature OBs, and this effect was observed to be concentration-dependent [69].

Peptides, which have high biocompatibility and specific tissue-targeting abilities, have long been used for several biomedical applications [70, 71]. In one study, to overcome some of the limitations of salmon calcitonin, such as its short half-life, it was loaded into pentapeptide-decorated silica NPs (SiO_2_-Pep@sCT). It was found that the alkaline phosphatase activity (ALP) of MC3T3-E1 cells was significantly higher (2 times and 1.4 times) in the SiO_2_-Pep@sCT group than in the SiO_2_ and salmon calcitonin groups, respectively, indicating that the external negative charge of the NPs and salmon calcitonin stimulated the differentiation of OBs. The number of calcified nodules was also higher in the SiO_2_-Pep@sCT group. In vivo bone parameters, such as BMD (1.2 times), the bone surface/bone volume ratio and bone volume/total volume ratio were all increased in the SiO_2_-Pep@sCT group. Therefore, silica NPs can be used as a potential drug carrier in OP and other skeletal diseases [72].

Mesoporous bioactive glasses (MBG) have been used for orthopedic and dental applications due to their strong bonding properties with the bone tissue. Moreover, they are biodegradable, which enables controlled drug release [73].

Estradiol (E_2_) use is a crucial therapeutic method for women with postmenopausal OP [74]. However, long-term treatment has disadvantages. In a previous study, mesoporous bioactive glass NPs (MBGNPs) were used to encapsulate E_2_. Moreover, they were modified with β-cyclodextrin (CD-MBGNPs) to improve their affinity for E_2_. These particles were then electrospun with silk fibroin (SF) to create a nanofibrous mesh (E_2_@CD-MBGNPs/SF). Further characterization of the NPs revealed a burst release in the first 48 h, followed by further release after day 13. The ALP activity of the cells (MC3T3-E1) was increased after treatment with E_2_@CD-MBGNPs/SF on day 7. In the case of OCs, the E_2_@CD-MBGNPs/SF group could reduce the total DNA amount, indicating the inhibiting nature of the nanofiber. Tartrate-resistant acid phosphatase (TRAP) activity was found to be significantly suppressed in the E2@CD-MBGNPs/SF group. Additionally, the presence of TRAP + multinucleated cells and the actin-ring formation was minimal in the E_2_@CD-MBGNPs/SF group versus the others. These observations all suggest that E_2_@CD-MBGNPs/SF is a potential anti-osteoporotic drug [75].

Cerium (Ce) is an element with diverse biological functions, including potent antioxidant properties that make it suitable for applications such as radiation protection, cardiovascular diseases, and neurological disorders [76–80]. In a previous study, mesoporous silica NPs used to encapsulate nanoceria (Ce@MSNs). Ce@MSNs were found to be toxic at concentrations exceeding 100 µg/mL but were internalized by MC3T3-E1 cells after 24 hours, allowing direct interaction with mitochondria to reduce oxidative stress. The ALP activity and calcification level of MC3T3-E1 cells were significantly increased in the Ce@MSNs group at 100 µg/mL compared to that of the MSNs group. Ce@MSNs were also found to decrease the number of multinucleated cells (RAW 264.7) when co-cultured with MC3T3-E1 cells, indicating their potential as anti-osteoclastic agents [81].

Long non-coding RNAs (lncRNAs) are a class of RNA with no protein-coding ability, and their role in bone formation is not well-studied. However, lnc-ob1 and ORLNC1 have been identified as crucial for bone formation [82, 83]. In a previous study, bioactive glass NPs (BGN) were used to induce bone marrow-derived mesenchymal stem cells (BM-MSCs) to secrete extracellular vesicles (EVs). It was reported that the EVs isolated after exposure to BGN successfully attenuated OC differentiation. Parameters associated with OC activity, such as the formation of multinucleated cells, TRAP activity, and the expression of *NFATc1*, were significantly downregulated after treatment with BGN-EVs [84].

#### Titanium nanotubes

Titanium nanotubes exhibit significant potential as materials for bone-tissue engineering, owing to their advantageous physicochemical properties that facilitate the adhesion and proliferation of OBs. Furthermore, they have been extensively investigated for their capacity to serve as carriers for osteogenic substances that encourage bone formation and enhance the differentiation of OBs. Promising outcomes from preclinical studies have demonstrated the exceptional osseointegration properties of these nanotubes, thereby establishing them as highly suitable contenders for orthopedic implants and therapies aimed at bone regeneration [85–88].

The primary methodology employed for fabricating titanium nanotubes is electrochemical anodizing. This process involves the preparation of titanium and platinum sheets, which are subsequently immersed in an electrolyte solution. The titanium sheet, acting as the substrate for nanotube growth, is connected to the positive electrode, while the platinum sheet is connected to the negative electrode. In this electrochemical configuration, the primary oxidation of titanium occurs at the anode, specifically at the interface between the metal and the oxide layer. Concurrently, reactions such as hydrogen evolution and oxygen reduction take place at the platinum electrode. By applying a continuous voltage, metal ions migrate outward through the oxide-electrolyte interface, while oxygen molecules migrate inward from the electrolyte-oxide interface to the surface of the metal-oxide. These migration processes result in the formation of titanium crystals, ultimately leading to the fabrication of titanium nanotubes [89].

Mu et al. utilized TiO_2_ arrays as carriers for raloxifene, and subsequently coated them with a hybrid multilayered coating composed of chitosan and alendronate-grafted hyaluronic acid (TNT/Ral/LBL-Aln). Through in vitro experiments, it was observed that TNT/Ral/LBL-Aln exhibited a twofold increase in ALP expression and a 1.4 times increase in mineralization. Additionally, it demonstrated the ability to inhibit OC differentiation. In vivo studies further demonstrated that the administration of raloxifene led to an increase in bone formation and a decrease in the level of TRAP activity, resulting in reduced trabecular space. These findings suggest that raloxifene not only suppresses OC differentiation but also enhances bone formation, thereby positioning it as a promising therapeutic option for OP [90].

Icariin (ICA), the primary active component found in Epimedium, has been identified as a promoter of bone formation. Furthermore, it has been observed to support fracture healing by mitigating oxidative stress [91]. Strontium (Sr) is another osteogenic component known for its exceptional biocompatibility [92]. In one particular study, a TiO_2_ nanotube surface was coated with Sr and ICA to investigate their effects on the cell adhesion, proliferation, and osteogenic differentiation of pre-OBs, as well as bone formation surrounding titanium implants. The group treated with TiO_2_ + Sr + ICA demonstrated a significantly higher proliferative and adhesive effect on MC3T3-E1 cells. Moreover, ALP activity increased by 1.7 times, and mineralization was found to be the highest in the TiO_2_ + Sr + ICA group, followed by the TiO_2_ + Sr and TiO_2_ groups. While new bone formation occurred around the implants in all three groups in vivo, the TiO_2_ + Sr + ICA group exhibited a greater bone volume relative to the total tissue volume (BV/TV) [93].

Calcitonin is a small peptide hormone that has been reported to inhibit OC-mediated bone resorption. Calcitonin also targets Wnt10b in OCs and promotes bone formation [94]. Studies have shown that calcitonin gene-related peptide (CGRP) immobilized onto TiO_2_ nanotubes (TNT-CGRP) can affect the differentiation of OBs and OCs in vitro. TNT-CGRP can indicate better cell adhesion on the implant, with better cell spreading and stress fiber formation. ALP activity was greatly noticed in the TNT-CGRP group, which was more than what was displayed in the other groups. In addition, mineralization was also observed to be the highest in this group. Osteogenic-related gene expression, such as collagen type I (*ColI*), Runt-related transcription factor 2 (*Runx2*), Osteopontin (*Opn*), and Osteoprotegerin (*Opg*), was greater in the TNT-CGRP group than in the Ti group in the 7-day culture. The mRNA levels of *Col1*, *Runx2*, *Opn*, and *Opg* were 3.0-, 2.2-, 2.1-, and 2.0-fold higher than those of the Ti group, respectively. In the case of OC differentiation, TRAP activity was lowest in the TNT-CGRP group in comparison with other groups. The expression levels of OC-related genes, such as vacuolar *(H+) ATPase*, *matrix metalloproteinase 9*, *TRAP*, and *cathepsin K*, were decreased after treatment with TNT-CGRP. Therefore, TNT-CGRP could improve bone formation while decreasing OC differentiation [95].

#### Hydroxyapatite NPs (HA NPs)

Hydroxyapatite (HA) is a natural inorganic material found in the bones and teeth of humans and vertebrates [96]. The chemical structure of HA is mainly known as Ca_10_(PO_4_)_6_(OH)_2_. HA is a biocompatible, osteoconductive, and bioactive ceramic widely recognized for its ability to establish direct bonds with living tissues. This property is attributed to its inherent high affinity towards collagen type I, making HA an exceptionally reliable candidate for bone formation [97]. In the field of bone regeneration, HA scaffolds and materials have been extensively utilized. Recently, HA nanoparticles have garnered attention due to their large surface area, which facilitates enhanced protein adsorption and cell interaction. The production of HA nanoparticles can be achieved through both dry-synthesis and wet-synthesis methods. Dry synthesis methods typically yield well-crystallized and stoichiometric products but require high temperatures and lengthy treatment times. On the other hand, wet-synthesis methods can produce nanoparticles at relatively low temperatures, but the resulting crystallinity and calcium-to-phosphorus (Ca/P) ratio are comparatively lower [98].

Zoledronic acid (ZOL), a bisphosphonate (BP) with anti-resorptive properties, is commonly employed in the treatment of OP [99]. In a recent study focused on investigating the potential synergistic effects of hydroxyapatite (HA) nanoparticles loaded with ZOL (HNLZ) on OP, promising results were obtained. In the HNLZ group, the serum levels of bone-specific ALP, procollagen type I N-terminal propeptide, osteocalcin, and TRACP 5b were significantly lower compared to those of the sham-operated group [100].

The potential of targeted delivery for OP has remained in its infancy. This study utilized salmon calcitonin (SCT)-loaded HA NPs (SCT-HAP-NPs) for the treatment of sublingual OP as a non-invasive therapy. It was observed that the SCT-HAP-NPs deeply penetrated the tissue through the stratified squamous epithelial layer via the basement membrane. Additionally, the SCT-HAP-NPs could significantly lower serum calcium and phosphorous levels followed by a significant decrease in the resorption pits [101].

Research has shown that targeted delivery of BP for OP is an area that requires further investigation. In a study using risedronate/zinc-hydroxyapatite NPs (ZnHA-NPs), it was found that ZnHA-NPs attenuated the increase in serum levels of bone-specific ALP and preserved the structural integrity of the cortical and trabecular bones. The study also showed that ZnHA-NPs successfully regulated the level of TRAP-5b, indicating the action of OC-mediated bone resorption [102].

Zhao et al. investigated the use of HA bioceramics composed of a micro whiskered scaffold strengthened with multiple layers of releasable HA NPs (nwHA) as a treatment option for OP. The study showed that the nwHA group induced new bone formation and had significantly higher expressions of the genes responsible for bone formation, ATP2A2 and FGF23, upon implantation in critical-sized femur defects in osteoporotic rats [103].

Hwang et al. tried to achieve the bone-specific dual delivery of both a drug and a mineral. To do this, hydroxyapatite nanoparticles (HA NPs) were modified with alendronate (Aln) and coated with multilayers of poly(allylamine) (PAA) and alginate (ALG). The resulting compound, Alen-LBL-HA NPs, was synthesized and investigated for its effects on bone-related cells. In vitro studies using MC3T3-E1 cells demonstrated that Alen-LBL-HA NPs exhibited high proliferation rates and significantly increased ALP activity. These findings suggest that Alen-LBL-HA NPs possess properties that enhance cell proliferation and promote bone formation [104].

Additionally, calcium-rich hydroxyapatite nanoparticles (CRHNPs) were examined for their impact on BM-MSCs in the context of OP. The study revealed that CRHNPs promoted the proliferation of BM-MSCs while reducing apoptosis. Furthermore, the expression levels of osteogenic markers, such as *Runx2* and *OPN*, were significantly increased by 1.6-fold and 1.5-fold, respectively, compared to those of the control groups. These results indicate that the use of HA NPs may hold promise as an approach for the treatment of OP [105].

### Metallic NPs

Tissue engineering extensively employs metal nanoparticles. Materials like Au, Ag, Fe, Al, Ni, Cu, Zr, and magnetic nanoparticles (MNPs) have been extensively investigated for this purpose. Although previous research highlighted the toxicity of certain metal NPs, such as Ni, it has now been established that when used in appropriate sizes and doses, metal NPs offer numerous benefits. Additionally, metallic NPs commonly possess desirable traits like high surface areas and antibacterial properties [106, 107].

#### Magnetic NPs (MNPs)

MNPs, which predominantly consist of iron oxide II and III, are composite crystals composed of magnetic elements such as Fe, Ni, or Co. Ni NPs exhibit desirable properties such as good Curie temperatures, chemical stability, and high saturation magnetization, making them excellent candidates for drug delivery systems. Cobalt ferrite NPs, on the other hand, possess exceptional mechanical hardness, wear resistance, ease of synthesis, and electrical insulation properties, which make them promising agents for various medical applications, including magnetic drug delivery. Additionally, MNPs can also serve as contrast agents for magnetic resonance imaging. Furthermore, MNPs can be utilized to induce thermolysis by exposing cells to radiofrequencies, offering a potential avenue for hyperthermia-based therapies [108–111].

Over time, diverse strategies have been developed to synthesize MNPs with control over their size, morphology, stability, and biocompatibility. These approaches can be broadly categorized into physical, chemical, and biological methods. Physical methods include techniques such as ball-milling, laser evaporation, and wire explosion. Chemical methods encompass coprecipitation, thermal decomposition, microemulsion synthesis, hydrothermal synthesis, and sol-gel methods. On the other hand, biological methods utilize the byproducts of plants or microorganisms and have gained recognition for their ability to synthesize MNPs [112].

Research has been conducted on fabricating BP-conjugated MNPs (BP/Dex/Fe_3_O_4_) to inhibit OC activation. Bis/Dex/Fe_3_O_4_ has a high binding affinity to bone grafts and decreases the activation of OCs by BP. Continuous radiofrequency exposure to BP/Dex/Fe_3_O_4_ induced thermolysis of OCs while having no effect on the survival of osteoblasts [113].

Tran et al. have shown that hydroxyapatite-coated Fe_3_O_4_ NPs (HA-IONP) significantly upregulates ALP, collagen, and calcium in OBs. The results indicate that HA-IONP promotes OB differentiation. The authors found that HA-IONP adsorbed a large amount of fibronectin and consequently enhanced the function of OBs and upregulated genes related to OB differentiation [114].

In another study, Li et al. developed HA-coated superparamagnetic iron oxide NPs (SPIO@HA) with a core − shell structure for targeting both osteoclastogenesis and osteogenesis. SPIO@HA exhibited chemical stability and low cytotoxicity in in vitro experiments. It promoted the differentiation of MSCs to OBs while inhibiting OC formation and downregulated genes related to osteoclastic differentiation. Additionally, SPIO@HA prevented bone loss and increased BMD in the OVX mouse model [115].

Extracellular vesicles (EVs) derived from MSC (MSC-EVs) can deliver therapeutic targets for various diseases [116]. Despite this, the isolation and detection of EVs still exhibit several technical drawbacks such as limited sensitivity and time consumption [117]. In a previous study, gold-coated magnetic nanoparticles (GMNPs) were used to load EVs. The surfaces of GMNPs were decorated with a Fe_3_O_4_@SiO_2_ core and a silica shell with PEG-aldehyde (CHO) to examine its role in diabetic osteoporosis (DO). Microarray analysis revealed that OP-associated miR-150-5p was differentially expressed. To establish models of OP, rats were injected with streptozotocin, and bone tissue analysis confirmed the reduced expression of miR-150-5p. Subsequently, a combination of GMNPs and anti-CD63 formed GMNP_E_, which was then co-cultured with OBs. The reintroduction of miR-150-5p facilitated osteogenesis in the OBs. GMNP_E_ played a role in enriching EVs in the bone tissues of the rats with OP. The miR-150-5p carried by BMSC-EVs targeted MMP14, thereby activating the Wnt/β-catenin pathway. This activation then enhanced the proliferation and maturation of OBs. Additionally, GMNP_E_ improved the delivery of miR-150-5p via EVs, effectively regulating the MMP14/Wnt/β-catenin axis and promoting osteogenesis [118].

#### Gold NPs

Gold NPs (GNPs) have been widely developed for therapeutic application of biologic therapies, including DDS of drugs and genes, photographic agents, photothermal therapies, biosensors, and diagnostic reagents. GNPs are highly influenced by the physical and chemical characteristics of their synthesis such as reaction temperature, stirring rate, and the ratio of gold to the reducing agent. To prepare GNPs, many researchers developed GNP fabrication methods such as green synthesis using plants or bacteria, the Turkevich-Frens method, Brust-Schiffrin method, Martin method, and Seeding-Growth method. GNPs have long been known as osteoinductive agents that inhibit OC formation [119–126]. High amounts of GNPs in the body can be toxic; therefore, it is necessary to modify the surface of these particles so that they reach their target sites.

BPs have been used as therapeutics against osteoporosis [127]. BPs, such as alendronate, have been conjugated with GNPs (GNPs-ALD) to specifically target OCs and inhibit their differentiation, and thereby, bone resorption. GNPs-ALD was also successful in inhibiting OC differentiation. It was found that 20 µM of GNPs-ALD fully inhibited the formation of RANKL-induced osteoclastogenesis. In addition, GNPs-ALD at 20 µM could inhibit the formation of TRAP + multinuclear cells, compared to the positive control group. The expression of OC-specific genes like *OSCAR, c-Fos*, and *NFATc1* were also significantly reduced when the cells were treated with 20 µM of GNPs-ALD. In addition, in vivo data showed that all OVX groups, except GNPs-ALD, had a lower trabecular bone volume [128].

**Fig. 2:**
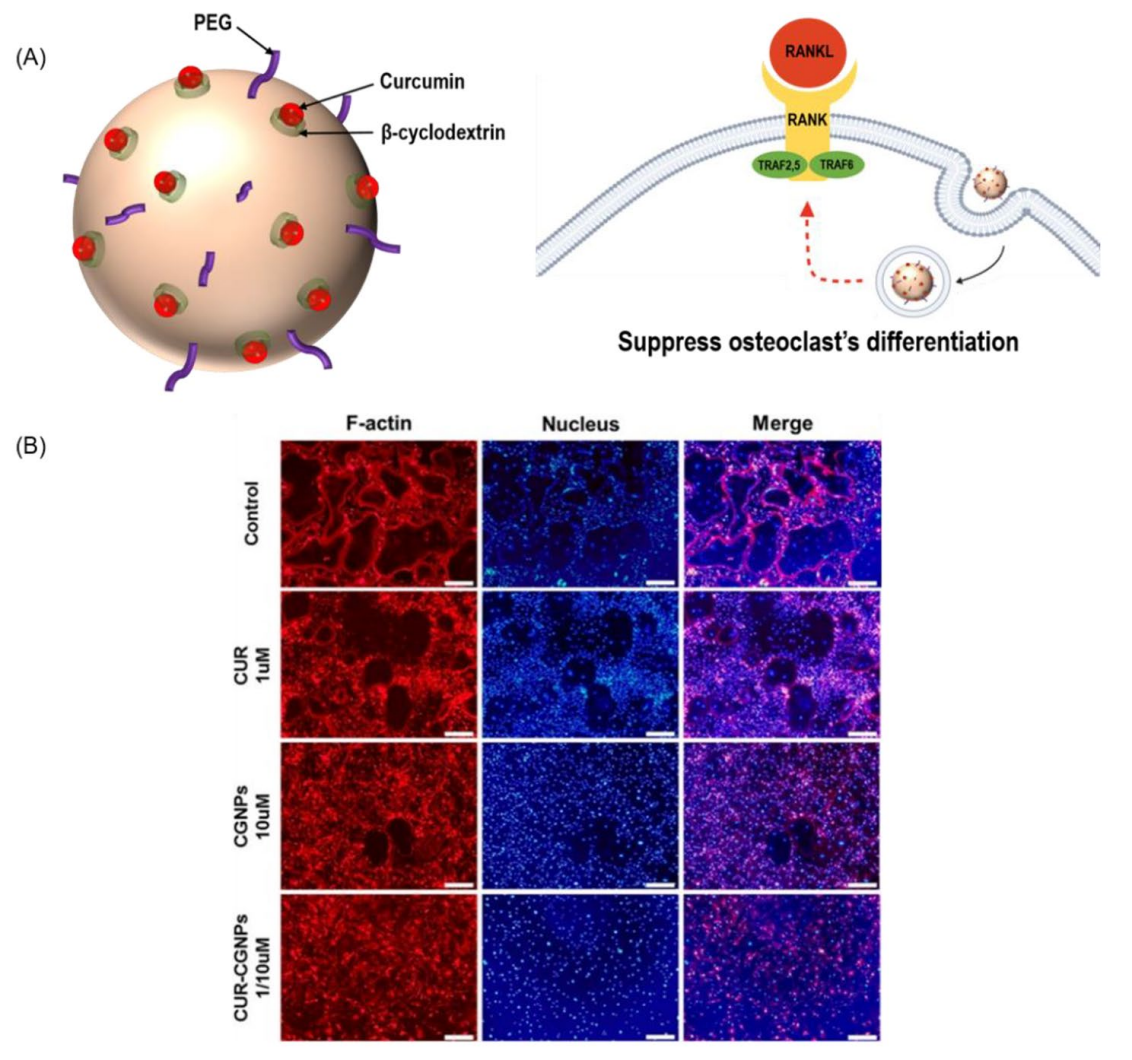
**Effect of CUR-CGNPs on OC**. (**A**) β-cyclodextrin-conjugated GNPs with curcumin inhibits the osteoclastic differentiation of bone-derived macrophages. (**B**) Immunofluorescence analysis of bone-derived macrophages incubated in osteoclastic differentiation medium with CUR, CGNPs, or CUR-CGNPs for F-actin expression (RANKL-induced actin-ring formation). GNP: Gold nanoparticles; BMSCs: Bone marrow derived mesenchymal stem cells; CUR: Curcumin; CGNPs: β-cyclodextrin-conjugated GNPs; CUR-CGNPs: Curcumin loaded β-cyclodextrin-conjugated GNPs; RANKL: Receptor activator of nuclear factor κB. (**B**) is reprinted (adapted) with permission from Heo, D.N., Ko, W.K., Moon, H.J., Kim, H.J., Lee, S.J., Lee, J.B., Bae, M.S., Yi, J.K., Hwang, Y.S., Bang, J.B., and Kim, E.C., 2014. Inhibition of osteoclast differentiation by gold nanoparticles functionalized with cyclodextrin curcumin complexes. ACS nano, 8(12), pp. 12,049–12,062. Copyright 2014 American Chemical Society

β-cyclodextrin-conjugated GNPs with curcumin (CUR-CGNPs) as an inclusion complex have been used to determine their effects on receptor activator of nuclear factor-κb ligand (RANKL)-induced osteoclastogenesis in bone-marrow–derived macrophages. In addition, CUR-CGNPs could significantly reduce the number of TRAP + multinuclear cells. The real-time PCR analysis showed that OC marker genes, such as *c-Fos*, *NFATc1*, and *OSCAR*, were also significantly downregulated after treatment with CUR-CGNPs (Fig. 2A). While RANKL stimulated actin-ring formation, which allows OCs to resorb bone, CUR, CGNPs, and CUR-CGNPs reduced actin-ring formation. Particularly, CUR-CGNPs showed almost no actin-ring formation, implying that CUR-CGNPs are the most useful therapeutic agents (Fig. 2B). In vivo data showed that CGNPs and CUR-CGNPs increased the bone density with a smaller trabecular number, in comparison with the OVX group. The BMD was also found to be high in the CUR-CGNPs treatment group [129].

Nah et al. investigated the effect of vitamin-D–conjugated GNPs (VGNPs) on RANKL-induced osteoclastogenesis. PEG-containing sulfhydryl groups were used to attach vitamin D to the surface of the GNPs. The VGNPs were successfully internalized by bone marrow macrophages, and it was found that 20 µM of VGNPs could decrease the formation of TRAP + multinuclear cells. Moreover, VGNPs downregulated genes related to OC differentiation, such as *TRAP*, *OSCAR*, *NFATc1*, and *c-Fos* [130].

The transcription factor c-myb is a member of the myeloblastosis (MYB) family and is crucial for cell differentiation, survival, death, and proliferation [131–133]. Several studies have investigated its role in osteogenesis and odontogenesis. In one study, chitosan-gold nanoparticles conjugated with plasmid DNA/c-myb (Ch-GNPs/c-myb) were found to upregulate c-myb expression and stimulate osteogenesis on titanium surfaces in MC3T3-E1 cells. Ch-GNPs/c-myb also inhibited OC differentiation in bone marrow-derived macrophages, significantly reducing the number of TRAP + multinucleated cells [134].

### Polymeric NPs

Polymeric nanoparticles exhibit superior stability within the gastrointestinal (GI) tract when compared to alternative colloidal carriers. This enhanced stability enables them to shield encapsulated drugs from the harsh conditions encountered in the GI environment. The utilization of diverse polymeric materials allows for the deliberate adjustment of physicochemical attributes, pharmacokinetic properties, and biological behaviors of nanoparticles. Furthermore, the surface of these particles can be conveniently modified through the adsorption or chemical grafting of specific molecules, such as polyethylene glycol (PEG), poloxamers, and bioactive compounds. These modifications offer opportunities to tailor the surface properties of NPs, thereby influencing their interaction with biological entities and improving their therapeutic potential [135, 136].

#### Poly lactic-co-glycolic acid nanoparticles (PLGA NPs)

PLGA, a hydrophobic biopolymer renowned for its exceptional biodegradability and biocompatibility characteristics, has emerged as a prominent candidate in the realm of biomedical applications. Remarkably, PLGA generates a biocompatible byproduct that can be efficiently eliminated via metabolic pathways, thereby bolstering its safety profile and rendering it a preferred choice in clinical therapeutic interventions. Functioning as an exemplary drug carrier, PLGA offers the ability to encapsulate a diverse array of pharmaceutical agents, thus conferring remarkable benefits such as enhanced bioavailability and sustained release of the encapsulated drug. This advantageous attribute holds significant importance and has propelled the widespread utilization of PLGA as a versatile platform for DDSs [137, 138].

PLGA NPs were synthesized using an emulsion method, specifically through two types of emulsions: (1) water/oil emulsion and (2) water/oil/water emulsion. Initially, PLGA powder was dissolved in an organic solvent such as chloroform, dimethyl sulfoxide, or dichloromethane. Subsequently, the PLGA solution was combined with a water-based solution, resulting in the formation of an emulsion. The mixed solution was then subjected to stirring while the organic solvent underwent evaporation. Finally, the remaining PLGA nanoparticles were obtained through the process of centrifugation [139].

The therapeutic efficacy of simvastatin (SIM)-loaded tetracycline-mediated PEG-PLGA (TC-PEG-PLGA) micelles was evaluated through their administration in osteoporotic rats, thereby delineating their potential impact. Intriguingly, the TC-PEG-PLGA micelles exhibited a remarkable augmentation in mineralization, with a remarkable two-fold increase observed in comparison to the negative control, specifically in MC3T3-E1 cells. Furthermore, the experimental findings unveiled that the administration of these micelles elicited a notable induction of osteogenesis in the rat model of osteoporosis. Remarkably, the bone mineral content of the rats subjected to TC-PEG-PLGA was significantly higher, with a 1.2-fold increase compared to that of the control groups, as documented in a previous study [140].

Gene delivery is an efficient method for treating OP [141]. In this approach, PLGA nanocapsules loaded with a PEI-RANK siRNA complex are used to suppress OC differentiation. The PLGA nanocapsules can be successfully internalized by RAW 264.7 cells, whose OC differentiation is consequently inhibited. Moreover, the nanocapsules can effectively downregulate *RANK*, leading to a significant reduction (50%) in the *RANK* mRNA level in OC precursor cells. The TRAP enzyme is also downregulated in the group exposed to the nanocapsules, indicating that PLGA nanocapsules loaded with a PEI-RANK siRNA complex can effectively inhibit OC differentiation [142].

17β- estradiol (E_2_) has several anabolic effects in maintaining the structural integrity of the bone. Postmenopausal OP is a serious issue among women for which there are very limited therapies [143]. In a study on transdermal DDSs, E_2_-loaded PLGA NPs were used for OP therapy. As E_2_ is affected by the first-pass hepatic metabolism, the study investigated the effect of iontophoresis on the dermal permeabilization of E_2_. It was found that the E_2_-loaded PLGA NPs significantly increased the dermal permeabilization of E_2_ upon applying iontophoresis. Furthermore, E_2_-loaded PLGA NPs increased the BMD of cancellous bone when compared with the OVX mouse control group [144].

#### Gelatin NPs

Gelatin is a promising material for various biomedical applications due to its biocompatibility, biodegradability, and non-toxic nature [145, 146]. In addition, using gelatin composites, such as NPs, microparticles, 3D scaffolds, and electrospun nanofibers, improved mechanical properties. Due to the release pattern of bioactive molecules, they can be controlled depending on the cross-linking density of gelatin. Gelatin NPs have been widely used as drug and gene carriers [147]. The most common method to fabricate gelatin NPs is cross-linking by glutaraldehyde (GA), genipin, and carbodiimide/N-hydroxysuccinimide. Briefly, gelatin and drugs in the aqueous phase (salt water or alcohol) are homogenized with the oil phase (olive oil, polymethyl methacrylate, or paraffin oil) and then cross-linked with GA or genipin. To collect gelatin NPs, water, and the oil solvent are removed by evaporation, filtration, centrifugation, and lyophilization [148].

**Fig. 3:**
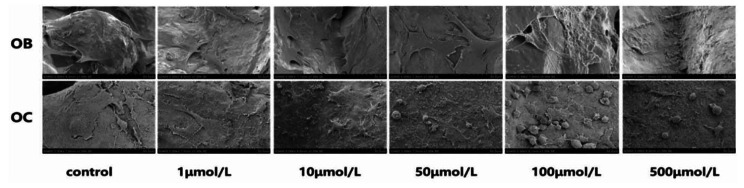
**SEM image showing the cell attachment and proliferation of OBs on different concentrations of ZOL-loading scaffolds**. OBs exhibited increased morphological elongation and filamentous filopodia at concentrations of 1 µmol/L to 50 µmol/L (Fig. 6). However, at concentrations of 100 µmol/L and 500 µmol/L, cell attachment to the scaffold decreased, resulting in atrophy and reduced pseudopodium formation. OB: Osteoblast; OC: Osteoclast. Reproduced with permission from Yang et al. [73]. (Copyright 2020, IOP Publishing Ltd.)

In a study conducted by Yang et al., a polydopamine-coated porous titanium scaffold was designed to be integrated with zoledronic acid (ZOL)-loaded gelatin NPs to investigate their effects on osteogenesis and osteoclastogenesis. From the findings illustrated in Fig. 3, it was discovered that OBs displayed a notable augmentation in their morphological elongation and the presence of filamentous filopodia when exposed to concentrations ranging from 1 µmol/L to 50 µmol/L. However, in the groups exposed to 100 µmol/L and 500 µmol/L, the number of cells adhering to the scaffold diminished, and they exhibited signs of atrophy and reduced pseudopodium formation. In addition, there was no significant difference in OC attachment to the scaffold at concentrations of 1–10 µmol/L. The osteogenic effect of ZOL-loaded NPs (ALP activity and expression of genes such as *Runx2* and *ALP*) was highest at 50 µmol/L, and the number of mature OCs (multinucleated cells) decreased at this concentration [149].

Strontium ranelate (SR) has the advantage of promoting bone formation and inhibiting bone resorption. However, high doses of SR may cause heart and kidney-related complications with frequent intake [150–152]. In a previous study, a cross-linking strategy was employed, which included enzyme-crosslinking using tyrosinase and physical folding to achieve SR-loaded gelatin NP/silk fibroin aerogel (S/G-Sr/MT). S/G-Sr/MT showed the highest level of ALP activity. In addition, the gene expression levels of the osteogenic markers *ALP, Runx2, Col1*, and *Osterix* were 10.0-, 6.0-, 1.5-, and 10.0-folds higher than those of the S/G group, respectively. Although the in vivo bone parameters (BV/TV, trabecular number [Tb.N], trabecular separation [Tb.Sp], and BMD) of the S/G-Sr/MT group were similar to those of the other groups, the S/G-Sr/MT group showed the highest bone union rate at each testing time point. Additionally, S/G-Sr/MT was also able to downregulate the activity of TRAP, a marker of bone resorption [153].

#### Chitosan NPs

Chitosan has been successfully used in many fields related to human health, pharmaceuticals, and the environment. Its biodegradability has paved the way for its numerous applications in various fields as a treatment option [154]. Furthermore, chitosan nanoparticles (NPs) play a significant role in protecting drugs from enzymatic degradation and minimizing adverse effects on non-targeted tissues or cells. These nanoparticles possess a positively charged surface and exhibit controlled and sustained drug release characteristics [155]. The fabrication of chitosan NPs commonly involves methods such as emulsification and crosslinking. Additionally, chitosan NPs can be prepared using alternative approaches including reversed micelles, phase inversion precipitation, and emulsion-droplet coalescence [156].

In a study on glucocorticoid-induced OP rats by Alshubaily et al., the anti-osteoporotic effect of Shilajit-loaded chitosan NPs was investigated. The study used nanochitosan (NCT) and NCT conjugated with a shilajit water extract (SWE) (NCT-SWE) as the main test groups. The results showed that NCT-SWE was very efficient in enhancing the levels of calcium, phosphorus, osteocalcin, and calcitonin. It was also successful in reducing hydrogen peroxide levels, thereby maintaining the level of antioxidants. Therefore, NCT-SWE could decrease oxidative stress and upregulate biomarkers of bone formation [157].

Santhosh et al. found that the treatment efficiency of BPs, such as risedronate, against osteoporosis was increased by functionalizing risedronate with chitosan NPs (RISCN). The results showed that RISCN has more affinity toward human farnesyl diphosphate synthase (FDPS) in the mevalonate pathway, thereby blocking the process of bone resorption by inhibiting osteoclastogenesis. Thus, RISCN is considered highly target-specific in treating OP [158].

BMP-2, a growth factor recognized for its ability to induce bone formation, faces limitations when administered as an injection due to its short half-life and poor retention efficiency [159, 160]. To address this challenge, researchers have developed an innovative approach using a dual-function injectable fibrin gel (Fg) combined with semisynthetic sulfated chitosan NPs (SCS-NPs) loaded with recombinant human BMP-2 (rhBMP-2). In a previous study, the Fg loaded with 20 mg of SCS-NPs and 5 µg of rhBMP-2 demonstrated a remarkable threefold increase in the gene expression of key markers for bone formation, including type 1 collagen (*Col-1*), *Osterix* (*Osx*), and *Runx2*. Moreover, this formulation exhibited accelerated bone formation in vivo [161].

#### Nanogels

Nanogels have been utilized as carriers for various drugs and are classified based on the bonds involved in polymer network formation. Chemically cross-linked nanogels are polymer chains that are covalently bonded, while physically crosslinked nanogels are formed by weaker interactions, such as hydrogen bonds, electrostatic interactions, and hydrophobic interactions [162].

Chemical crosslinking serves as the primary approach for synthesizing nanogels, encompassing techniques like emulsion polymerization, controlled/living radical polymerization, click chemistry, and photo-crosslinking. This method involves utilizing low molecular weight monomers, short polymers, or polymer precursors with reactive end groups. Initiating materials, such as initiators, catalysts, and crosslinking agents, play a crucial role in the process. In nanogel formation, radicals are generated by the initiator through various means such as heat, hydrogen ions, or light. These radicals initiate reactions with monomers or polymers, resulting in the formation of monomer radicals or polymer radicals. Subsequently, the monomer or polymer radicals engage with other monomers or polymers, establishing covalent bonds. Furthermore, radicals derived from previously formed covalent bonds continue the chain of reactions, leading to the creation of additional covalent bonds. Ultimately, this radical-induced polymerization reaction culminates in the formation of nanogels. In contrast, the physical crosslinking method for nanogel formation relies on supramolecular polymers or biomolecules. These materials facilitate the spontaneous aggregation or self-assembly of nanostructures without the necessity of crosslinking agents. The driving forces in this process include ionic and hydrophilic-hydrophobic interactions, as well as Van der Waals and hydrogen bonds. Notably, during the formation of physically cross-linked nanogels, properties such as size, morphology, and strength undergo alterations influenced by factors such as ionic strength, temperature, and pH [163, 164].

Glucocorticoid-induced OP is the most prevalent cause of secondary OP, where exposure to glucocorticoids worsens the risk of fracture and bone loss. In one study, a transdermal nanoemulsion gel formulation was developed, and lovastatin was loaded into the nano-sized globules of the nanoemulsion to promote skin layer entry while avoiding liver metabolism. It was discovered that biomechanical strength testing indicated better strength and load-bearing capacity, and anabolic markers for bone formation were also found to be elevated, while levels of bone resorptive markers decreased [165].

Zhang et al. developed a combination nanogel scaffold (N.E) fabricated from p(N-isopropylacrylamide-co-butyl methacrylate) and a strontium MBG scaffold (Sr-MBGS). N.E can retain large amounts of water and harden at body temperature, making it suitable for transplantation into living cells. The combination of N.E and Sr-MBGS particles was found to serve as an excellent delivery vehicle for primary OBs delivered directly to the scaffold site for tissue regeneration [166].

Nanogel-mediated peptide delivery has also been investigated as a treatment option for bone loss. In one study, a cholesterol-bearing pullulan (CHP) nanogel was used to encapsulate the W9-peptide, TNF-α, and RANKL antagonist. A decrease in BMD was significantly prevented in the CHP-W9 peptide-injected groups. Additionally, histomorphometric analysis of the proximal tibiae showed a significant prevention in changes to bone resorption parameters, such as a decrease in the number of mature OCs [167].

Guo et al. synthesized a raloxifene HCL-loaded solid NP (RAL-SLNs) decorated gel to alleviate the effects of OP. The successful loading of RAL-SLNs into the gel allowed for its retention for a long period of time, along with the convenience of transdermal delivery. Biochemical analysis of the ALP and calcium levels in OP-induced rat models showed that the levels were significantly higher after treatment with RAL-SLNs [168].

#### Polyurethane nanomicelles

Polyurethane, a polymer composed of organic units linked by carbamate bonds, offers excellent biocompatibility, low cytotoxicity, and mechanical flexibility, making it ideal for drug encapsulation and long-term stability [169]. In addition, polyurethane can form a micelle structure through its self-assembly property in an aqueous solution. Thus, the polyurethane micelles are composed of a hydrophilic shell and a hydrophobic core, allowing them to be used as a DDS for encapsulating either hydrophilic or hydrophobic drugs. Also, polyurethane micelles can have a target effect on specific tissues by surface modification with antibodies and peptides [170, 171].

**Fig. 4:**
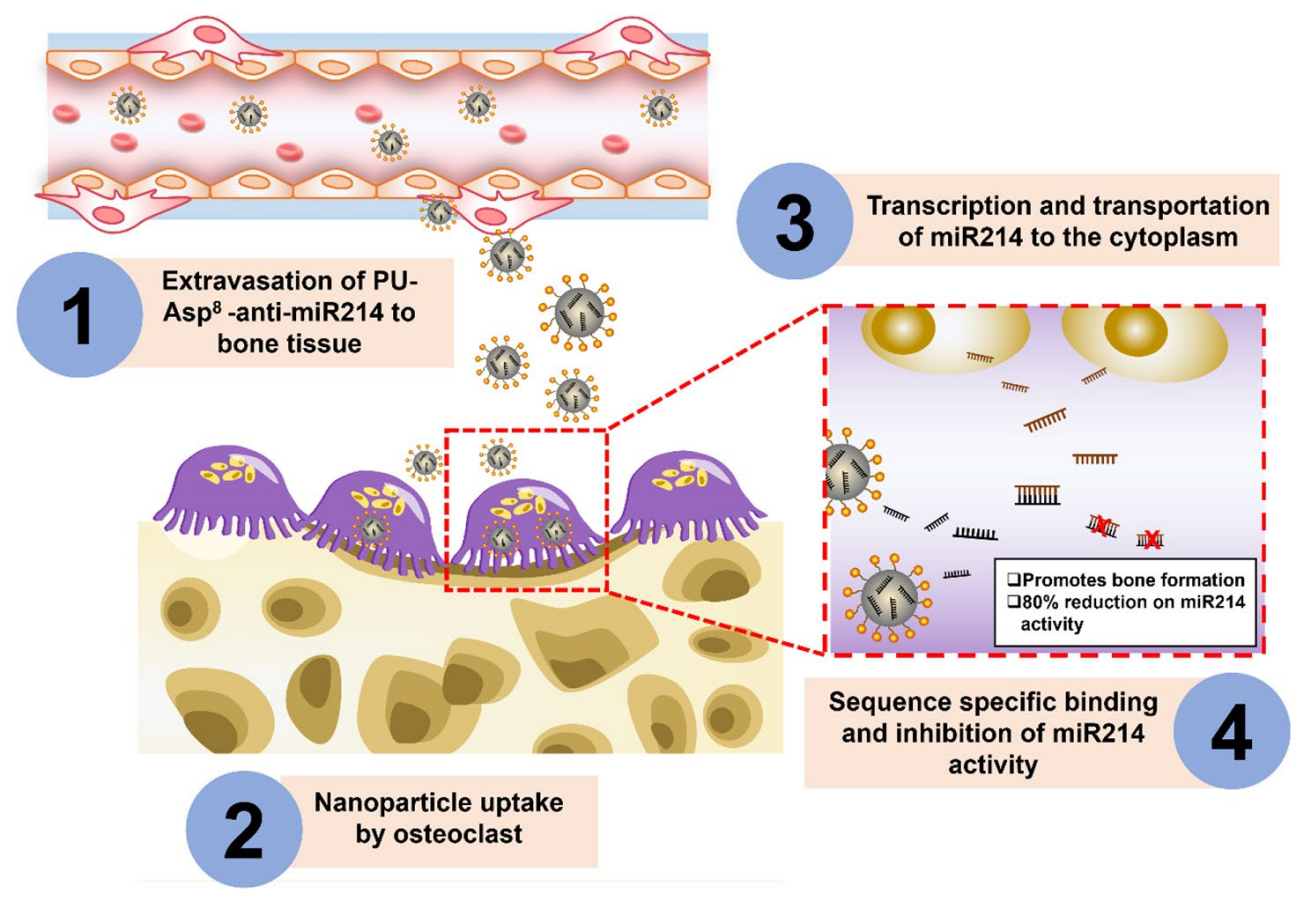
**PU-ASP**^**8**^**-modified polyurethane nanomicelles targeting OCs**. ASP^8^ has a higher affinity towards crystallized hydroxyapatite surfaces (resorbed bone surface). After the PU-ASP^8^ reaches the bone microenvironment, it undergoes cellular uptake by OCs, leading to an 80% reduction in the expression of miR-214. This substantial decrease in miR-214 levels plays a crucial role in improving bone health by promoting an increase in bone mass and a simultaneous decrease in trabecular space. PU-polyurethane; ASP^8^-eight repeating sequences of aspartate; OC- osteoclast

Peptide targeting is a promising approach in its early stages of development. Researchers have used polyurethane nanomicelles modified by the peptide ASP^8^ (PU-ASP^8^) to deliver anti-miR214 specifically to OCs, reducing miR214 efficiency by 80%, increasing bone mass, and decreasing trabecular spacing with an increase in BMD (Fig. 4) [172]. Similarly, SDSSD-modified polyurethane (PU) nanomicelles encapsulating anti-miR-214 were developed to target OBs, which resulted in an 80% reduction in miR-214 levels in OBs after SDSSD-PU-anti-miR-214 treatment. Targeting specific cell types in OP treatment may be a promising avenue for future research to improve drug targeting efficiency. SDSSD-PU-anti-miR-214 was also successful in improving the bone mass density along with inducing a higher mineral apposition rate with high miRNA stability [173].

### Lipid-based NPs

Lipid-based nanocarriers have gained attention as potential vehicles for OP drugs due to their high biocompatibility, biodegradability, and ability to release drugs in a controlled manner after various routes of administration [174–178]. Liposomal formulations and solid-core micelles are the most widely studied lipid-based NPs, with surface modifications improving their therapeutic outcomes such as long-circulation, tissue-targeted effect, and pH-sensitivity. The different approaches used for the fabrication of lipid-based NPs are high-pressure homogenization, emulsion, solvent evaporation, solvent diffusion, and ultrasonication [179].

In a study investigating the effect of quercetin on bone health, quercetin-based solid lipid NPs (QSLNs) were formulated. QSLNs were found to restore trabecular and bone mineral density in ovariectomized mice compared to the control group. Additionally, QSLNs significantly downregulated the osteoclastogenic genes *RANK*, *TRAP*, and *c-Fos*, indicating the potential of these NPs as anti-osteoporotic therapeutics [180].

**Fig. 5:**
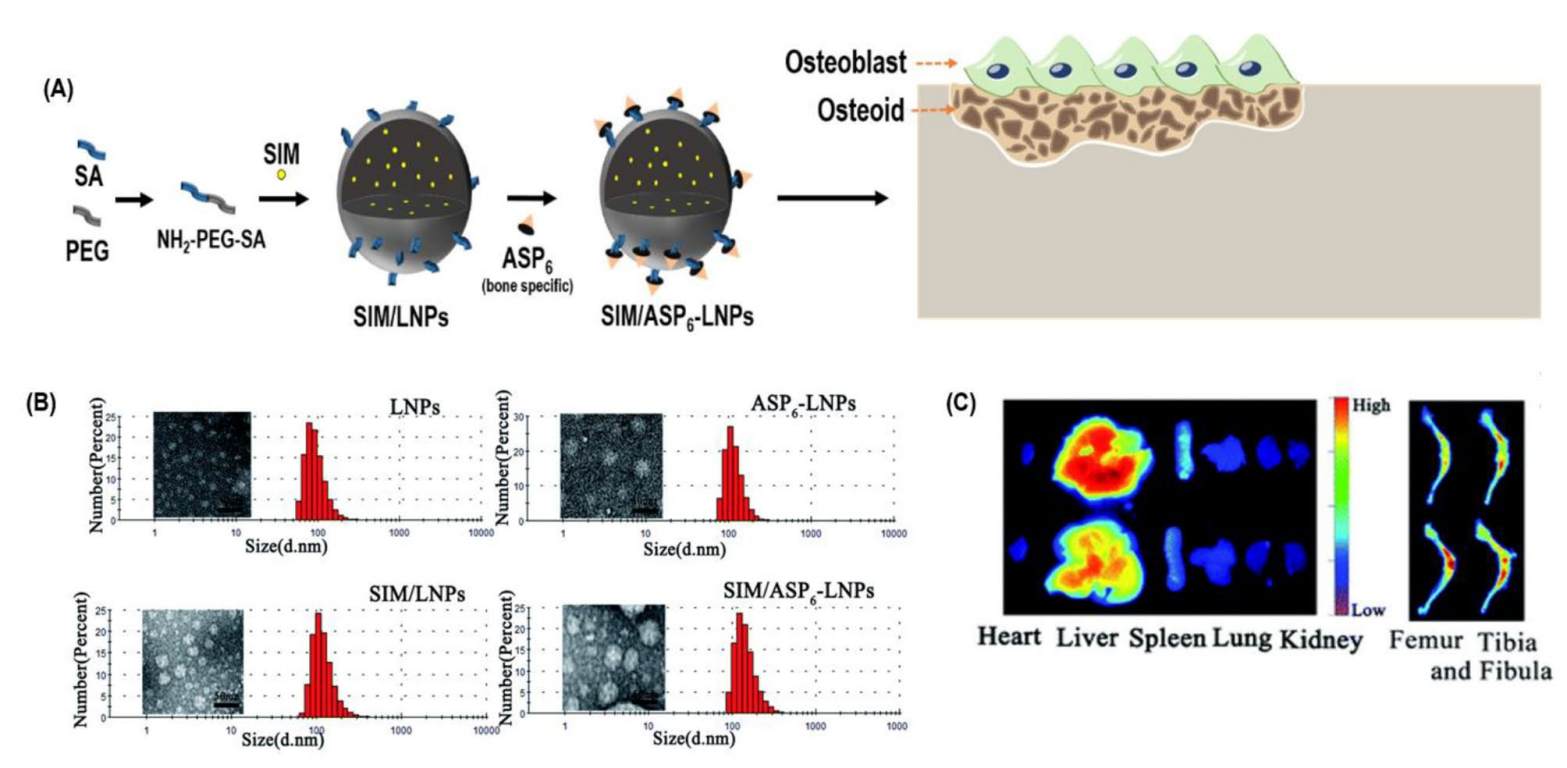
**A schematic presentation of the improved efficacy of simvastatin-loaded LNPs in treating osteoporosis**. (**A**) SIM was enclosed within lipid nanoparticles (NPs) that were further modified with aspartic oligopeptide 6 (ASP_6_) moieties. The purpose of incorporating ASP_6_ onto the NPs was to facilitate targeted delivery and enhance the affinity of the resulting formulation, known as SIM/ASP_6_-LNPs, towards bone formation processes. (**B**) Characterization of the LNPs, ASP_6_-LNPs, SIM/LNPs, and SIM/ASP_6_-LNPs. Left: TEM images. Right: size distribution based on DLS. Scale bar: 100 nm. (**C**) Distribution of DiR-loaded LNPs and ASP_6_-LNPs in ICR mice. Fluorescence image of the major organs (the heart, liver, spleen, lung, and kidney) and the femur and tibia 48 h after the tail-vein administration of the nanoparticles. LNPs: Lipid NPs; ASP_6_-LNPs: Lipid NPs with aspartic oligopeptide; SIM/LNPs: Lipid NPs delivering SIM; SIM/ASP_6_-LNPs: SIM encapsulating LNPs with ASP_6_; TEM: Transmission electron microscopy; DLS: Dynamic light scattering; DiR: Tetramethylindotricarbocyanine iodide; ICR: Institute of Cancer Research. (**B**) and (**C**) were reproduced with the permission from Tao et al. [111]. (Copyright 2020, The Royal Society of Chemistry)

Simvastatin (SIM) is commonly used to treat high cholesterol levels. However, recent studies have suggested that SIM can increase bone formation through BMP-2 [181]. In a study investigating the effect of SIM on bone formation, SIM was encapsulated in lipid NPs with aspartic oligopeptide 6 (ASP_6_) moieties, which were grafted onto the NPs to target bone formation (SIM/ASP_6_-LNPs) (Fig. 5A). The formation of mineralized nodules between SIM/LNPs and SIM/ASP_6_-LNPs indicated that SIM was responsible for the mineralization in both groups at an equal concentration of 10^− 7^ M. Furthermore, 29% of tetramethylindotricarbocyanine iodide (DiR)-loaded ASP_6_-LNPs were found in the femur and tibia, suggesting the bone-targeting specificity of the NPs. In vivo bone parameters such as BMD and BV/TV were increased in comparison with the sham-operated group, indicating the potential of SIM/ASP_6_-LNPs to target bone and promote its formation (Fig. 5B) [182].

Cathepsin K is an enzyme secreted by OC to digest collagen and other bone-matrix proteins. Inhibiting or neutralizing the expression of cathepsin K may suppress bone resorption [183]. In one study, the cathepsin K inhibitor, odanacatib, was loaded into PLGA-derived lipid hybrid NPs, which were modified with bone-specific polyaspartic acid [(ASP)_8_ DNPs] to investigate its effect on OP. Here, the binding affinity of (ASP)_8_-DNPs was two times higher than that of the DNPs. Odanacatib-loaded (ASP)_8_-DNPs were found to have the least TRAP activity and could downregulate OC-related genes, such as *Cathepsin K*, *TRAP*, and *RANK*. Along with inhibiting OC activity, osteogenesis-related genes, such as *Runx2* and *ALP*, were also upregulated after the treatment with odanacatib-loaded (ASP)_8_-DNPs. Several in vivo bone parameters such as BMD and BV/TV were higher following the odanacatib-loaded (ASP)_8_-DNPs treatment than with the odanacatib-loaded DNPs treatment [184].

**Fig. 6:**
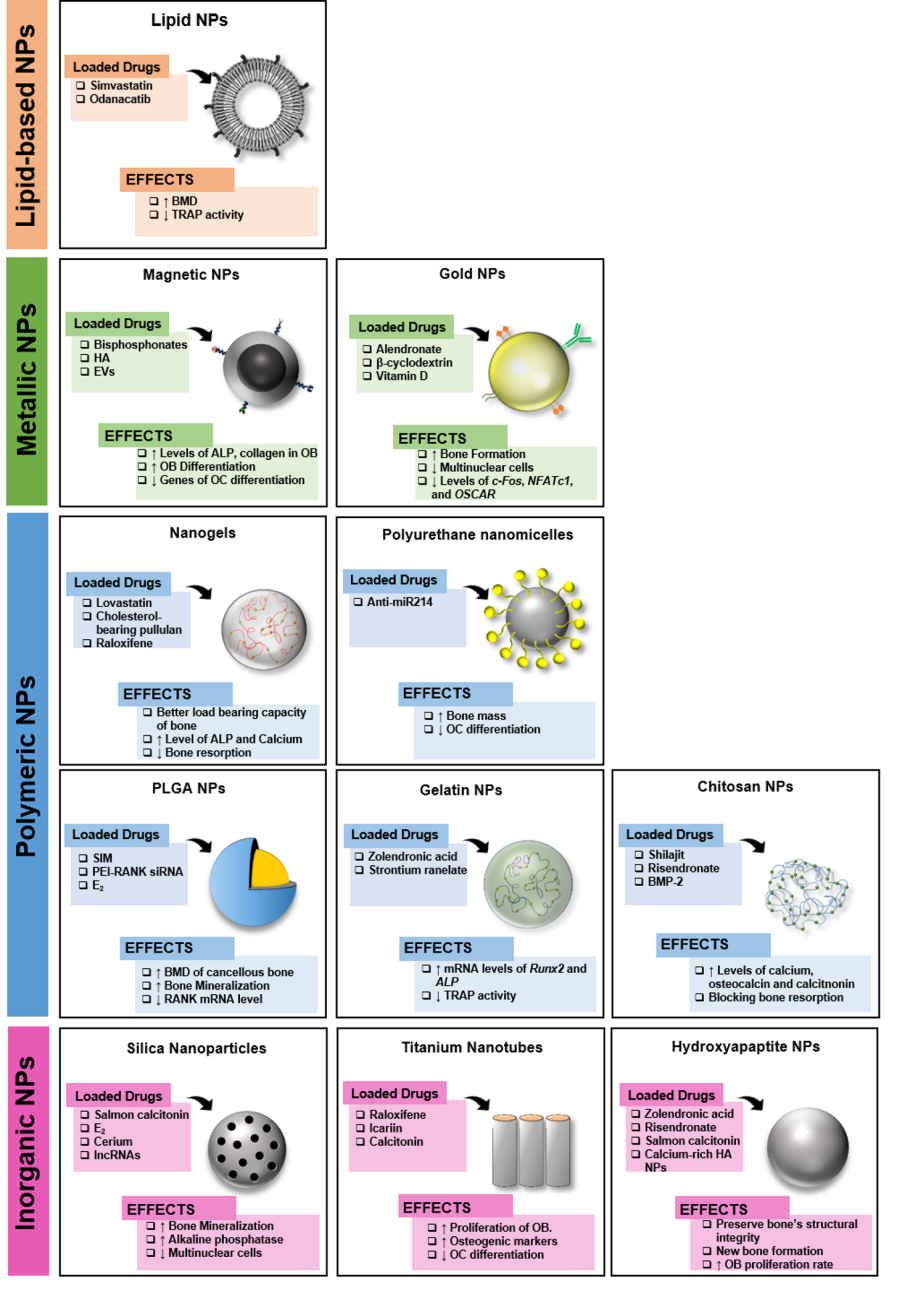
**A schematic representation of different drug-loaded nanocarriers and their effects**. An extensive repertoire of NP formulations, encompassing diverse categories such as inorganic, metallic, polymeric, and lipid nanoparticles, presents a versatile array of options for loading pharmacological agents aimed at combatting OP. These NPs, functioning as potent drug carriers, facilitate precise and targeted delivery, thereby conferring therapeutic benefits that encompass the dual capacity to foster bone formation or impede OC differentiation, thus mitigating the deleterious effects of OP. NP: Nanoparticles; RANK: Receptor activator of nuclear κB; EVs: Extracellular vesicles; HA: Hydroxyapatite; NFATc1: Nuclear factor of activated T-cells; BMP-2: Bone morphogenetic protein; SIM: Simvastatin; BMD: Bone mass density; TRAP: Tartrate-resistant acid phosphatase; OC: Osteoclast; OB: Osteoblast; ALP: Alkaline phosphatase; lncRNA: Long non-coding ribonucleic acid

Prior scientific investigations have conclusively demonstrated that MSCs possess the remarkable ability to perceive signals from damaged tissues and exhibit directed migration towards the site of injury. This migration process is facilitated by the intricate interplay between chemo-attractant stromal cell-derived factor 1 (SDF-1) and its specific receptor, chemokine receptor type 4 (CXCR4). Notably, experimental inhibition of CXCR4 has been observed to effectively impede the migratory response of MSCs. Harnessing the inherent homing and engraftment capacities instigated by the SDF-1/CXCR4 axis holds great promise for therapeutic applications in regenerative medicine and tissue repair [185–187]. In a previous study, the BMSC secretomes were loaded into PLGA NPs and were further modified by encapsulating the NP inside the CXCR-overexpressed HMEC membrane (MSC-Sec/CXCR4 NPs). MSC-Sec/CXCR4 NPs at a concentration of 10^6^ were able to successfully inhibit RANKL-induced OC differentiation simultaneously, promoting the osteogenic capacity and proliferation of MSCs. The levels of osteocalcin and BMP-2 were found to increase in the OVX rat model. Injection with MSC-Sec/CXCR4 NPs increased the bone volume while decreasing bone resorption [188]. An overall explanation of the nanocarrier types with their drugs along with their effects have been described in Table 2. The impact of different nanocarriers loaded with various drugs on bone has been illustrated in Fig. 6.

**Table 2:** **Effects of several nano-based drug delivery systems in treating osteoporosis.** In nano-based DDSs, the bare NPs can affect bone regeneration by forming a bone matrix or promoting mineralization. In addition, drug-encapsulated NPs can increase the stability of the drug in vivo and decrease side effects from excessive drug delivery. Recently, there have been many studies for the bone tissue targeting of NPs by conjugation and hybridization with peptides, BPs, and lipid-based NPs.

Ref. No.	Material	Type of carrier	Drug	Effects
[69, 72, 75, 81, 84]	Silica NPs	Silica NPs	-	Biocompatibility, increased bone mineral density
		pentapeptide (GGGGD)-decorated silica NPs	Salmon calcitonin	Increased circulation time and loading efficiency, enhanced bioavailability, sustained release, biocompatibility, biodegradation, avoids the immune system
		β-cyclodextrin-modified MBGNPs	17β-estradiol	Sustained drug release, promoted osteogenesis, deposited HA-like layer (Si^2+^, Ca^2+^, and P^5+^)
		Mesoporous silica NPs	Ceria	Stimulated bone forming of OBs and suppressed OC differentiation, modulated deposited HA-like layer solution (Si^2+^)
		Bioactive glass NPs (60SiO_2−_36CaO_4−_P_2_O_5_)	lncRNA NRON	Induced production of extracellular vesicles enriched in lncRNAs inhibiting OC differentiation, enhanced bioactivity and biocompatibility
[90, 93, 95]	Titanium nanotube	O_2_-anodized titanium nanotubes with chitosan/alendronate/hyaluronic acid layers	Raloxifene	Sustained drug release, strong binding of bone minerals to the titanium implant, promoted bone formation
		O_2_-anodized titanium nanotubes	Icariin and strontium	Sustained drug release, biocompatibility
		O_2_-anodized titanium nanotubes with polydopamine coating	Calcitonin gene-related peptide	Enhanced bioactivity and biocompatibility
[100–105]	Hydroxyapatite NPs	HA NPs	Zoledronic acid	Increased bone formation
		HA NPs	Salmon calcitonin	Enhanced bone targeting and penetration of the mucosa layer
		Zinc-HA NPs	Risedronate	Enhanced bone targeting, improved bone properties
		Multilayer of releasable HA NPs	HA	Bioceramics; highly promoted bone regeneration, reinforced mechanical performance, good potential as a bone graft
		Alendronate-modified HA NPs	Alendronate	Enhanced bone targeting, and increased proliferation of pre-OB
		Calcium-rich HA NPs	Calcium	Promoted the osteogenic differentiation of BM-MSC
[113–115, 118]	Magnetic NPs	Bisphosphonate-conjugated magnetic NPs	Bisphosphonate	Suppressed OC activation
		H-coated magnetic NPs	HA	Increased adsorbing of fibronectin, promoted osteoblastic differentiation
		HA-coated superparamagnetic NPs	HA	Promoted OB differentiation, inhibited OC differentiation, downregulated expression of genes related to osteoclastic differentiation, prevented bone loss, increased bone mineral density
		gold-coated magnetic NPs	MSC-EVs containing miR-150-5p	Activated the Wnt/β-catenin pathway, enhancing the proliferation and maturation of OBs.
[128, 129, 131, 134]	Gold NPs	Gold NPs	Alendronate	Enhanced biocompatibility and biostability, no cytotoxicity or genotoxicity, strong bone-surface affinity, stimulated osteoblastogenesis, and suppressed osteoclastogenesis
		β-cyclodextrin–conjugated gold NPs	Curcumin	Stimulated osteoblastogenesis and suppressed osteoclastogenesis, can carry hydrophobic drugs, increased solubility and stability
		Gold NPs	Vitamin D	Suppressed osteoclastogenesis, increased uptake by macrophages
		Chitosan-modified gold NPs	c-myb gene	Increased DNA stability, suppressed osteoclastogenesis
[140, 142, 144]	PLGA NPs	Tetracycline-PLGA micelles	Simvastatin	Enhanced bone targeting, increased proliferation of pre-OBs, and increased circulation time
		PLGA nanocapsules	PEI-RANK-siRNA complex	Increased siRNA stability
		PLGA NPs	Estradiol	High dermal permeability, improved bone mineral density in the bone
[149, 153]	Gelatin NPs	Gelatin NPs	Zoledronic acid	Increased drug stability, enhanced drug loading via electrostatic interactions, sustained and stable drug release, avoids phagocytosis
		Gelatin NPs/silk fibroin aerogel	Strontium Ranelate	Sustained drug release, controlled drug degradation, enhanced biocompatibility, suitable mechanical properties
[157, 158, 161]	Chitosan NPs	Chitosan NPs	Shilajit	Antioxidant bioactivity, decreased the oxidative stress
		Chitosan NPs	Risedronate	Enhanced biocompatibility and bone targeting and reduced therapeutic dose
		Chitosan NPs	BMP-2	Stimulated osteoblastogenesis
[165–168]	Nanogel	Nanoemulsion gels	Lovastatin	Increased biological permeability
		PIB nanogel scaffolds	Strontium-loaded mesoporous bioactive glass	Sol-gel transition-dependent temperature, enhanced drug release
		CHP nanogels	W9 peptide	Prevented peptide aggregation, increased peptide stability
		Nanogels	Raloxifene-HCl–loaded solid lipid NPs	Enhanced permeation and bioavailability
[172, 173]	Polyurethane nanomicelles	ASP^8^-modified nanomicelles	miRNA	Good biocompatibility and encapsulation efficiency, increased miRNA stability, enhanced bone targeting
		Pentapeptide (SDSSD)-modified nanomicelles	siRNA	Good biocompatibility and encapsulation efficiency, increased siRNA stability, enhanced osteoblast targeting
[180, 182, 184, 188]	Lipid-based nanocarriers	Solid lipid NPs	Quercetin	Effective multiple delivery routes (oral, intravenous, pulmonary, and transdermal), enhanced bioavailability and drug solubility
		ASP_6_-modified lipid NPs	Simvastatin	Bone targeting, enhanced biocompatibility and bioavailability, prevented drug degradation by the extracellular environment, increased water solubility, high drug-loading efficiency, large-scale production
		ASP_8_-modified lipid-coated PLGA NPs	Odanacatib	Bone targeting, enhanced biocompatibility and bioavailability, prevented drug degradation by endogenous enzymes, high drug-loading efficiency, large-scale production
		Human microvascular endothelial cell membrane- coated PLGA NPs	MSC secretome	inhibited OC differentiation while promoting osteogenic proliferation

NP: nanoparticle; EVs: Extracellular vesicles; PLGA: Poly lactic-co-glycolic acid; NRON: ncRNA repressor of the nuclear factor of activated T cells; PIB: Poly(N-isopropylacrylamide-co-butyl methylacrylate); CHP: Cholesterol-bearing pullulan; ASP: Aspartic acid oligopeptide; OB: Osteoblast; OC: Osteoclast; HA: Hydroxyapatite; MSC: Mesenchymal stem cell; BMP: Bone morphogenetic protein

## Discussion

OP is a skeletal disorder that worsens with age, making it crucial to develop new delivery systems to tackle the issue with maximum efficiency. Application of biomaterials for bone regeneration is transforming the lives of patients by reducing off-target effects and increasing therapeutic efficiency. New methods for drug delivery are being developed worldwide, offering promising drugs through material-based DDSs.

Anti-osteoporotic drugs aim to rebalance bone metabolism by promoting OB differentiation or inhibiting OC differentiation. This review explained several nano DDSs that are based on targeted or non-targeted approaches for the treatment of OP. Materials such as Bis, Sr, and HA have been formulated in combination with other bone-targeting molecules to increase the efficiency of osteogenesis. In some cases, bone-targeting peptides together with other osteoconductive materials are used for efficient targeting with fewer side-effects. Oral administration of several osteoporotic drugs leads to poor bioavailability and drug retention in the body. However, targeted bone therapy eliminates these problems to a certain extent. This review shed light on different types of nano-based DDSs. Although these methods are well-established by in vitro and in vivo studies, further research is still needed.

Numerous advances have been made in the field of nano-based drug delivery for OP.

- Enhanced targeting strategies: Surface modifications, such as the use of ligands or antibodies, enable active targeting of specific cells or receptors in osteoporotic bone, improving drug delivery efficiency.
- Controlled and sustained release: This allows for prolonged drug exposure and therapeutic effects, reducing the frequency of drug administration.
- Combination therapies: The co-delivery of multiple therapeutic agents, such as anti-resorptive drugs and bone-stimulating factors, within a single nanocarrier provides synergistic effects and comprehensive treatment of osteoporosis.
- Advanced imaging and diagnostics: NPs with imaging capabilities have been developed, enabling non-invasive monitoring of drug distribution, bone health, and treatment response. These imaging techniques provide valuable insights for personalized treatment optimization.
- Smart nanocarriers: These are nanocarriers that respond to specific stimuli, such as pH changes or enzymatic activity in the bone microenvironment. These nanocarriers can be engineered to release drugs precisely at the desired sites, enhancing therapeutic effectiveness.
- Theranostic approaches: This integrates both therapeutic and diagnostic functionalities. These systems can simultaneously deliver therapeutic agents while providing real-time imaging and monitoring the treatment response.
- Biomimetic nanocarriers: These mimic the body’s inner environment, offering improved biocompatibility and facilitating targeted drug delivery to osteoporotic bone.

## Conclusion and future perspectives

OP is a bone-associated disorder characterized by a loss of bone mass and increased susceptibility of fractures. Currently, the primary treatment for osteoporosis is the use of drugs to slow the rate of bone loss or promote bone formation. However, these drugs are inadequate in their performances with serious long-term side effects.

Nano-based DDSs have the potential to revolutionize the treatment of OP. These systems include the use of nanoscale particles to deliver the drug to the bone. By using this system, drugs can be delivered to specific sites within the body with increased precision and efficacy, reducing side effects and improving patient outcomes. In the future, nano-based DDSs for OP may become the preferred method for treating this condition.

In the context of translating scientific findings from basic research into practical applications and solutions, the goal is to ensure the safer administration of drugs into the body system. In pre-clinical studies, the stability, drug release kinetics, and targeting efficiency of the nano-based DDSs in cell cultures and animal models of OP are assessed. Once a promising nano-based delivery system is identified, its pharmacokinetic properties like absorption, distribution, metabolism, and excretion from the body are studied. Next, the safety and toxicity evaluation is conducted to investigate its side effects and adverse effects. The nano-based DDS is then subjected to clinical study involving experimentation on human participants with OP after which the researchers seek regulatory approval from health authorities, such as the Food and Drug Administration (FDA). After its approval, the nano-based DDS can be commercialized and made available to the patients.

Even though the different delivery systems in this review have been well researched, certain challenges still persist for their clinical translation. Some of those are mentioned below.

- Safety concerns: NPs should be thoroughly evaluated for their potential toxicity, biocompatibility, and long-term effects on various organs or tissues.
- Scale-up and manufacturing: Scaling up the production of nano-based DDSs while maintaining quality control and reproducibility can be challenging. Cost-effective manufacturing processes need to be developed for large-scale production and commercialization.
- Targeting specificity: Achieving precise targeting of NPs to bone tissues while minimizing off-target effects remains a challenge. Improving the specificity and selectivity of NP accumulation in osteoporotic bone is crucial for optimizing therapeutic outcomes.
- Pharmacokinetics and biodistribution: Understanding the pharmacokinetics, clearing mechanisms, biodegradation pathways, and biodistribution of NPs in the body is important for determining the optimal dosing regimen and predicting potential side effects.
- Clinical translation: Bridging the gap between preclinical studies and clinical translation is a significant challenge. Comprehensive and well-designed clinical trials are needed to assess the safety, efficacy, and long-term effects of nano-based delivery in human patients.
- Immunogenicity and immune response: Immunological reactions may impact the efficacy of the delivered drugs and the overall safety profile of the nano-based delivery system.
- Regulatory considerations: Complying with regulatory requirements and obtaining necessary approvals for nano-based DDSs is a crucial step in their clinical translation. Meeting regulatory standards for safety, efficacy, and quality control for their successful implementation in patient care is necessary.

By addressing these challenges, nano-based drug delivery systems for OP can contribute to therapeutic advancements, offering more targeted, safe, and effective treatments for patients. Some of the possible suggestions are listed below.

- Improved bone targeting: Further research is needed to enhance the specificity and selectivity of nanocarriers for bone targeting. Exploring new targeting ligands or receptors specific to osteoporotic bone can improve the accumulation of NPs at the desired sites.
- Combination nanotherapies: Investigating synergistic combinations of NPs, including drugs, gene therapies, or stem cell therapies, can lead to more efficient treatment approaches by targeting multiple aspects of OP.
- Personalized medicine: Integrating personalized medicine approaches, such as genetic profiling or individualized bone quality assessments, can guide the optimization of nano-based DDSs. Tailoring treatments to individual patient characteristics can improve treatment efficacy and minimize adverse effects.
- Clinical translation and validation: Conducting well-designed clinical trials is essential for validating the safety, efficacy, and long-term effects of the delivery system in human patients. Close collaboration between researchers, clinicians, and regulatory agencies is necessary for successful clinical translation.
- Patient compliance and convenience: Developing delivery systems like long-acting implants or transdermal patches can enhance treatment adherence and simplify administration procedures.
- Bioinformatics and computational modeling: Utilizing these models to predict and optimize the behavior of nano-based delivery systems can guide the design process and accelerate the development of effective therapies.

**Fig. 7:**
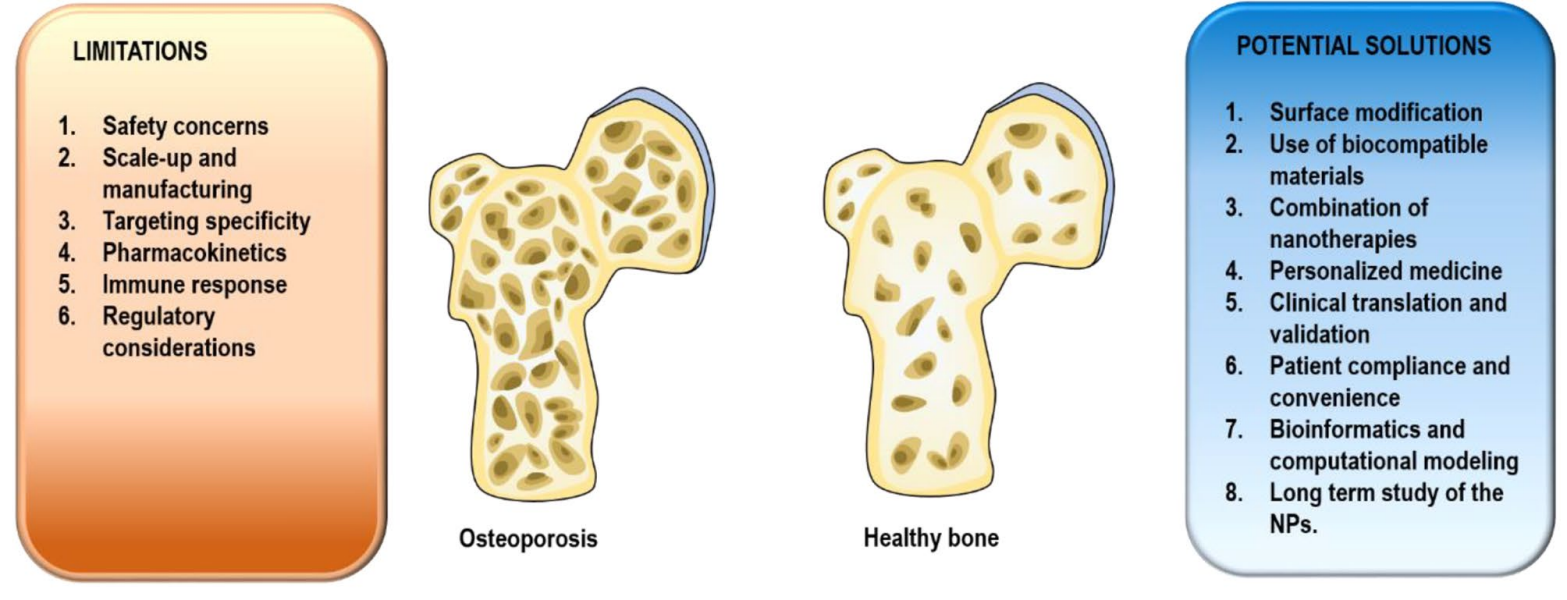
**Limitations of nano-based drug delivery systems and their potential solutions**. Nano-based drug delivery systems have shown immense potential in revolutionizing drug delivery for various medical conditions, including osteoporosis. However, they are not without their limitations such as safety concerns and immune responses. It is crucial to identify these limitations and explore potential solutions to overcome them for the successful implementation of nano-based DDSs. NPs-Nanoparticles

In conclusion, nano-based DDSs hold great promise for addressing the challenges associated with OP treatment. The unique properties of nanomaterials, such as their small size, controlled release capabilities, and targeted delivery potential, offer exciting opportunities for enhancing therapeutic outcomes in this debilitating condition. By utilizing nanotechnology, it is possible to improve the bioavailability, stability, and efficacy of therapeutic agents for OP. Moreover, nano DDSs have the potential to minimize off-target effects, reduce systemic toxicity, and enable personalized medicine approaches. Even though there are still many limitations associated with nano-based DDSs, the solution can be achieved by more innovative designs and extensive studies, as highlighted in Fig. 7. While further research and development are needed, the advancements in nano-based drug delivery for OP offer a hopeful path towards more effective treatments and improved quality of life for patients.

## Electronic supplementary material

Below is the link to the electronic supplementary material.

Supplementary Material 1

## Data Availability

Not applicable.

## Abbreviations

ALG: Alginate
Aln: Alendronate
ALP: Alkaline phosphatase
ASP: Aspartic oligopeptide
ATP2A2: Sarcoplasmic/endoplasmic reticulum calcium ATPase2
BP: Bisphosphonate
BMD: Bone mineral density
BM-MSCs: Bone marrow-derived mesenchymal stem cells
BMP: Bone morphogenetic protein-2
CD: β-cyclodextrin
Ce: Ceria
CGRP: Calcitonin gene-related peptide
CRHNPs: Calcium-rich hydroxyapatite nanoparticles
CUR: Curcumin
CXCR4: Chemokine receptor type 4
DDSs: Drug delivery systems
DNA: Deoxyribonucleic acid
DO: Diabetic osteoporosis
E2: 17β estradiol
EPR: Enhanced permeation and retention
EVs: Extracellular vesicles
FDPS: Farnesyl diphosphate synthase
FGF23: Fibroblast growth factor 23
GI: Gastrointestinal tract
HA: Hydroxyapatite
HMEC: Human microvascular endothelial cells
HNLZ: Hydroxyapatite nanoparticles loaded with zoledronic acid
ICA: Icariin
lncRNAs: Long non-coding RNAs
MBGNPs: Mesoporous bioactive glass nanoparticles
MBGS: Mesoporous bioactive glass scaffold
MNP: Magnetic nanoparticle
MSC: Mesenchymal stem cell
MYB: Myeloblastosis
NCT: Nanochitosan
NPs: Nanoparticles
OB: Osteoblast
OC: Osteoclast
OP: Osteoporosis
OVX: Ovarectomized
PAA: Poly allylamine
PEG: Polyethyelene glycol
PEI: Polyethylenimine
PLGANPs: Poly lactic-co-glycolic acid
QSLNs: Quercetin-based solid lipid nanoparticles
Ral: Raloxifene
RANKL: Receptor activator of nuclear factor kappa-B ligand
RhBMP-2: Recombinant human bone morphogenetic protein-2
RNA: Ribonucleic acid
SCT: Salmon calcitonin
SDF-1: Stromal cell-derived factor 1
SF: Silk fibroin
SIM: Simvastatin
SPIO@HA: Hydroxyapatite-coated superparamagnetic iron oxide
Sr: Strontium
SWE: Shilajit water extract
Tb.N: Trabecular number
Tb.Sp: Trabecular separation
TC: Tetracycline
TEOS: Tetraethoxysilane solution
TiO_2_: Titanium dioxide
TNT: Titanium dioxide nanotubes
TRAP: Tartrate-resistant acid phosphatase
VGNPs: Vitamin-D-conjugated gold nanoparticles
ZOL: Zoledronic acid
