# Supplementary material for: Emerging nano-scale delivery systems for the treatment of osteoporosis
Source: Biomater Res. 2023 Jul 13;27:68. doi: 10.1186/s40824-023-00413-7 (PMC10347748; doi:10.1186/s40824-023-00413-7)
Supplement: Supplementary file 1 — Supplementary Material 1 [file 40824_2023_413_MOESM1_ESM.pdf]

EDITORIAL CERTIFICATE

Date: Jul 02, 2023

Manuscript Author(s): Soo-Hong Lee

Manuscript Title: Emerging nano-scale delivery systems for the treatment of osteoporosis

To Whom It May Concern:

This letter confirms that the manuscript corresponding to the information detailed above was edited by a professional, native English-speaking editor at Wordvice.

We guarantee 100% language accuracy in the text, as edited and delivered to the author(s) on the date below. We make no claims as to the substantive matter covered by the paper and have not altered the intent or research content drafted by the author(s).

The author(s) may accept or reject any of our comments or suggestions upon receipt of the document we edited. Should you have any questions or concerns, please contact Wordvice at edit@wordvice.com

Sincerely,
Wordvice

Wordvice is a premier international English editing service. Our mission is to help researchers, scholars, students, and professionals reach their full potential through clear communication in their writing. By providing premium English editing services at affordable rates to clients from around the world, Wordvice seeks to tear down language barriers and contribute to the advancement of research and education.

Signature

Kevin Heintz
Managing Editor, Wordvice

Date of Issue
Jul 02, 2023

Contact Wordvice

KOREA	U.S.	JAPAN	TAIWAN	CHINA	TURKEY
edit@essayreview.co.kr	edit@wordvice.com	edit@wordvice.jp	edit@wordvice.com.tw	edit@wordvice.cn	edit@wordvice.com.tr
1522-9180	www.wordvice.com	+81 03-4500-8427	070-1017-6093	95013-5301-2128	+90 0530 390 6216
www.essayreview.co.kr		www.wordvice.jp	www.wordvice.com.tw	www.wordvice.cn	www.wordvice.com.tr
